# Performance of ^68^Ga-labeled prostate-specific membrane antigen ligand positron emission tomography/computed tomography in the diagnosis of primary prostate cancer: a systematic review and meta-analysis

**DOI:** 10.1590/S1677-5538.IBJU.2020.0986

**Published:** 2021-03-05

**Authors:** Xianwen Hu, Yan Wu, Peiqing Yang, Ju Wang, Pan Wang, Jiong Cai

**Affiliations:** 1 Affiliated Hospital of Zunyi Medical University Department of Nuclear Medicine Zunyi Guizhou China Department of Nuclear Medicine, Affiliated Hospital of Zunyi Medical University, Zunyi, Guizhou, China

**Keywords:** Prostate-Specific Antigen, Prostatic Neoplasms, Positron Emission Tomography Computed Tomography

## Abstract

**Objective:**

To explore the feasibility of ^68^Ga-PSMA PET/CT in diagnosing primary prostate cancer.

**Materials and Methods:**

Embase, PubMed and Cochrane Library databases were searched for studies published before July 2020. The studies that used ^68^Ga-PSMA PET/CT for detecting primary prostate cancer, and pathological biopsy as the reference standard were included. The selecting process used preferred reporting items for systematic reviews and meta-analyses (PRISMA). The quality of enrolled studies was assessed by the Quality Assessment of Diagnostic Accuracy Studies-2 (QUADAS-2) tool.

**Results:**

According to our search strategy, 9 studies were included for analysis. A total of 547 patients with primary prostate cancer and 443 lesion segments that underwent ^68^Ga-PSMA PET/CT scans were included and their pathological biopsies were compared. The results of these studies showed some differences. For instance, the lowest sensitivity of ^68^Ga-PSMA PET/CT in diagnosing primary prostate cancer was 67%, while the highest sensitivity recorded was 97%.

**Conclusions:**

Compared with conventional imaging examinations, ^68^Ga-PSMA PET/CT had higher sensitivity and specificity in detecting primary prostate cancer. At present, most of the studies that used ^68^Ga-PSMA PET/CT for detecting prostate cancer are retrospective studies. Based on its advantage of high detection rate, the use of ^68^Ga-PSMA PET/CT in the detection of primary prostate cancer is worthy of promotion.

## INTRODUCTION

Prostate cancer poses a serious threat to the health of men all over the World and its detection rate is increasing year by year with the development of medical technology. Imaging plays an important role in early and accurate diagnosis of prostate cancer (1). Conventional imaging tests for prostate cancer diagnosis include ultrasound, computed tomography (CT), magnetic resonance imaging (MRI), 2-deoxy-2-[fluorine–18]fluoro-D-glucose positron emission tomography/computed tomography (18F-FDG PET/CT), etc. Previous studies have demonstrated high specificity of transrectal ultrasound biopsy and multi-parameter MRI in diagnosing prostate cancer. However, the sub-rectal ultrasound biopsy has increased the risk of overdiagnosis and overtreatment of indolent prostate cancer (2), and the sensitivity, accuracy and specificity in diagnosing prostate cancer by multi-parameter MRI varied greatly (3).

The physical half-life of positron radionuclide ^68^Ga prepared by ^68^Ge/^68^Ga generator is 67.71 min. The positron decay accounts for 89% of the decay process, and the remaining 11% involves electron capture, considering that it is suitable for the pharmacokinetic study of small-molecular drugs and labeling of radioactive tracers (4). More than 90% of patients with prostate cancer have had high expression of prostate-specific membrane antigen (PSMA) in their cell membranes (5), and thus it might be an ideal drug target to treat radiation. Based on the above characteristics, ^68^Ga-labeled PSMA has been successfully developed as a nuclear medicine clinically, and has been reported in the diagnosis of liver cancer and kidney cancer in addition to prostate cancer (6, 7). In recent years, ^68^Ga-PSMA PET/CT was proved to be highly sensitive and specific in diagnosing prostate cancer. But, whether ^68^Ga-PSMA PET/CT completely replaces invasive biopsy by transrectal ultrasound in diagnosing prostate cancer in the future has become a hot research topic (8). Therefore, a meta-analysis and systematic review was conducted to evaluate the diagnostic performance of ^68^Ga-PSMA PET/CT in detecting prostate cancer.

## MATERIALS AND METHODS

According to the preferred reporting items of the systematic review and meta-analysis (PRISMA) guidelines, the present meta-analysis was conducted. The studies that compared the diagnostic performance of ^68^Ga-PSMA PET/CT in primary prostate cancer with histopathology were included.

### Literature search strategy

In July 2020, a systematic search of the EMBASE (including MEDLINE), PubMed, and Cochrane Library databases was conducted according to the PRISMA guidelines. The search criteria were as follows: ([“^68^Ga-labeled prostatic membrane antigen”] or [“^68^Ga-PSMA”] or [“gallium- PSMA”] or [“gallium-68 prostatic membrane antigen”]) and ([“PET/CT”] or [“Positron Emission Tomography-Computed tomography”] or [PET] or [“Positron Emission Tomography”]) and ([“prostat* neoplasm”] or [“prostat* cancer”] or [“prostat* carcinoma”] or [“prostat* tumor”]) and ([detection] or [detectability] or [positivity] or [accuracy] or [diagnosis] or [specificity] or [sensitivity] or [performance]). The search was limited to human studies, and in vitro and animal studies were excluded. As no patients were included in this study, informed consent or ethical review board (ERB) approval was not required.

## STUDY SELECTION PROCESS

### Inclusion criteria

The inclusion criteria were as follows: studies (I) that used The inclusion criteria were as follows: studies (I) that used ^68^Ga-PSMA PET/CT for diagnosing primary prostate cancer; (II) that used histopathological examination as reference standard for comparison; (III) in which prostate cancer was confirmed by biopsy or postoperative histopathological examination; (IV that included true positive, false positive, true negative, and false negative data to construct a 2x2 quadrilateral contingency table; (V) with at least 10 patients; and (VI) that were originally published.

### Exclusion criteria

The exclusion criteria were as follows: (I) study population with metastatic or recurrent prostate cancer (however, if the study provides diagnostic performance for each stage of prostate cancer including primary prostate cancer should be included); (II) review articles, case reports/series, diagnostic guides, short surveys, letters, consensus statements, study registrations, and conference summaries; (III studies with less than 10 patients; (IV) if the data included in the study was insufficient to construct a 2x2 quadrilateral table; and (V) ^68^Ga-PSMA PET/CT was used for diagnosing prostate cancer, but its diagnostic performance was not emphasized.

The establishment and literature selection process of this study was conducted by two independent authors with more than 10 years of scientific research experience. In case of any disagreements, consensus was reached by consulting the reviewers.

### Data extraction

The following data were extracted from the included studies in a standardized form: (I) characteristics of patients-number of patients, median age, median value of prostate specific antigen, Gleason score median and range; (II) features of studies included- origin of research (first author, nation), year of publication, study design (prospective or not, multicenter or not), reference criteria, blinding to reference criteria, and (III) PET/CT characteristics-PET/CT manufacturers, minimum scan layer thickness, radioactive tracer dose, uptake time, and CT technique and the mean maximum standard uptake value (SUVmax) for lesion images collected from the patients with PET/CT.

Quality assessment of diagnostic accuracy studies-2 (QUADAS-2) was used for assessing the quality of enrolled studies (9). Data extraction and critical evaluation were carried out independently by two authors, and any disputes between them were resolved by reaching a consensus with the third reviewer.

### Data integration and analysis

The primary purpose of this meta-analysis was to evaluate the diagnostic performance of ^68^Ga-PSMA PET/CT in diagnosing primary prostate cancer. Secondly, the heterogeneity between the included studies was also analyzed and attempted to explore its underlying causes.

True positive, false positive, false negative, and true negative data of ^68^Ga-PSMA PET/CT in diagnosing primary prostate cancer were extracted from the included studies (some of the data were deduced according to the paper) to make a 2x2 quadrellar contingency table, and the sensitivity and specificity were also calculated. If the included studies were given grades according to the prostate-specific antigen (PSA) values, then the detection rate was synthesized from it. When multiple reference indicators such as PSA value, maximum standard uptake value and Gleason score were given, then the results with the highest accuracy were used. The hierarchical logistic regression model was used to calculate the general estimates of sensitivity and specificity of the included study, which included the hierarchical summary receiver operating characteristics (HSROC) model and concomitant variables. HSROC curves with 95% confidence and prediction regions were used to map the results for their sensitivity and specificity. Cochran's Q test and Higgins I2 test were used to examine their heterogeneity. In Cochran's Q test, p <0.05 was taken as the test standard, which indicated the existence of heterogeneity. Higgins I2 test was used to evaluate the degree of heterogeneity using the following criteria: inconsistency index (I2) <50% was considered as heterogenous; I2=50-80% represents the possibility of moderate heterogeneity, and I2>80% represents the possibility of significant heterogeneity. Stata software 14.0 was used for statistical analysis, and p <0.05 was considered to be statistically significant.

## RESULTS

### Studies and study design

**Figure 1:**
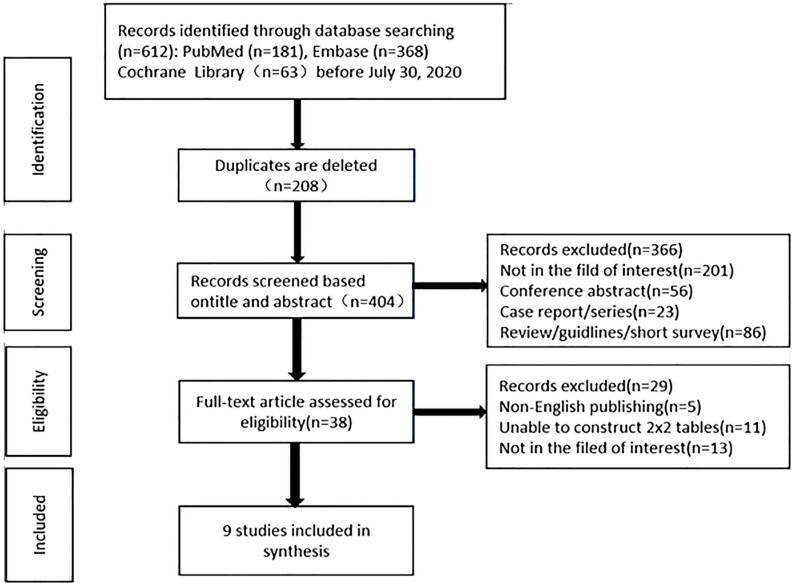
The literature screening flow chart of the inclusion study.

Literature search initially yielded 612 articles that diagnosed prostate cancer by ^68^Ga-PSMA PET/CT. After gradually removing the overlapping, irrelevant, reviews, case reports and other articles, there were 38 potentially eligible original texts. Due to non-English publications (n=5), the 2x2 quadrilingual table (n=11) could not be constructed by the data extracted from these papers, and the papers that were not the field of interest (n=13) were further excluded. Finally, 9 papers were included for meta-analysis, and the detailed process of literature search is presented in Figure-1.

The characteristics of patients in the enrolled studies (10–18) are presented in Table-1. Seven of the 9 included studies assessed the diagnostic performance of ^68^Ga-PSMA PET/CT for prostate cancer based on the number of patients (a total of 547 patients) and 3 studies (one of the article included studies on both patients as well as lesion segments) evaluated the diagnostic performance based on lesion segments (a total of 443 lesion segments). The study population size ranged from 24 to 173, and the average age, PSA values and Gleason scores were recorded from 8 of the 9 studies. The patients included in this study presented with clinical symptoms, had increased PSA, or pathologically confirmed prostate cancer.

**Table 1:** Features of the included studies.

First Author, Nation, Ref.	PY	Sample size(n)	Mean age	Mean PSA ng/mL	GS		Study design		RS	Blind	Vendor	STmin mm	RTH (mBq)	UT (min)	CT technique	Mean SUVmax
					Mean	Range	PD	MC								
Fendler et al., Germany (10)	2016	126(lesion)	NR	NR	7	6-9	Yes	No	biopsy	NR	Siemens	5.0	104-276	45-80	Non-CE and CE	11.8
Woythal et al., Germany (11)	2018	31	67.2	17.49	NR	6-10	No	No	biopsy	Yes	Philips	4.0	117.23± 19.86	60.90±26.13	Non-CE	14.06
Lopci et al., Italy (12)	2020	168(lesion)	74.7	7.6	NR	6-10	Yes	No	biopsy	Yes	NR	NR	NR	NR	NR	4.29
Chandra et al., India/(13)	2019	41	69.1	13.7	8	6-10	No	No	biopsy	NR	GE	NR	NR	NR	Non-CE	8.53
Kallur et al., India (14)	2017	76	68	25.5	7	5-9	No	No	biopsy	Yes	GE	3.75	M=111 R=74-185	60	Non-CE and CE	>4.0
Donato et al., Australia (15)	2020	144 (149,lesion)	67	8.6	7	6-8	No	No	biopsy follow-up	NR	Siemens	NR	150±7.5	60	Non-CE	NR
Basha et al., Egypt (16)	2019	173	68	17.7	8	6-10	Yes	Yes	biopsy	Yes	Philips	NR	NR	NR	NR	NR
Sachpekidis et al., Germany (17)	2016	24	69	24.1	7	7-9	No	Yes	biopsy	Yes	Siemens	NR	M=268 R=69-352	55-60	Non-CE	14.3
Zhang et al. China (18)	2019	58	70	15.46	NR	6-10	No	Yes	biopsy	Yes	Siemens	5.0	1.8–2.2 MBq/kg	60	Non-CE	8.76

**Notes: Ref** = Reference; **PY** = Publication year; **M =** Median; **R** = Range; **NR** = Not reported; **STmin** = Minimum slice thickness; **CT** = Computed tomography; **GS**=Gleason score; **PD** = Prospective design; **MC** = Multicenter; **RS**= Reference standard; **CE** = Contrast-enhanced; **Non-CE** = None contrast-enhanced; **RTD** = Radioactive tracer dose; **UT** = Uptake time.

Three of the 9 studies were conducted in Germany, 2 in India and the others in Italy, Australia, Egypt and China, and were published from 2016 to 2020. Among these studies, 3 studies were prospective studies, and the remaining were retrospective studies. Pathological tissue biopsy was used as the reference, and 5 of the 9 studies were blinded with the reference criteria.

Four studies were scanned by Siemens, 2 by GE, 2 by Philips PET/CT scanners, and the other one was not recorded. Four of the 9 studies recorded minimum thickness of the scan, and 6 studies recorded the dose of radioactive tracer and uptake time, while 2 studies did not record the CT technique used and the maximum standard uptake value of the lesion.

### Quality evaluation

**Figure 2:**
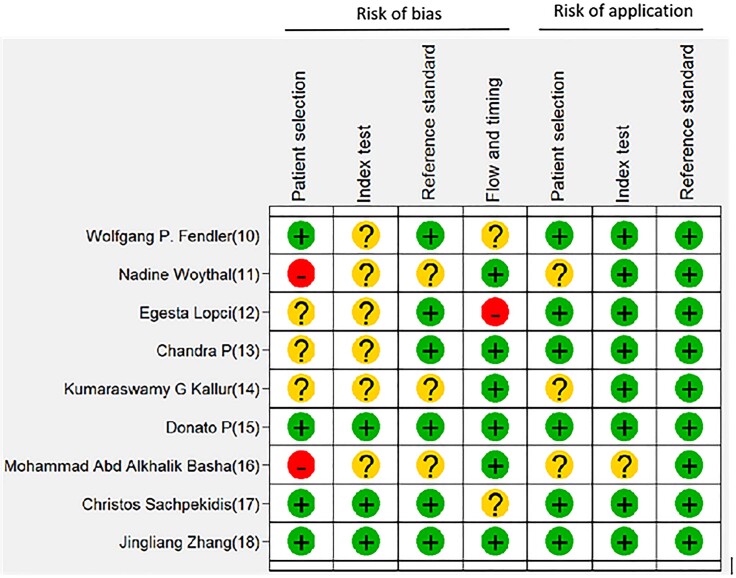
Risk of bias and applicability concern summary: Review the authors’ judgments of each of the areas covered in the study by QUADAS-2 (Quality Assessment of Diagnostic Accuracy Studies-2 tool).

The QUADAS-2 tool was used to evaluate the quality of the studies included as shown in Figure-2. In an objective assessment, the quality of these studies was shown to be medium to high, and 6 of the 9 studies met at least 4 of the 7 QUADAS-2 indicators. Of the 6 retrospective studies, one had high risk of bias due to discontinuous study design (11), and one retrospective study did not explicitly mention whether the patient registry was continuous or not (16), and so the risk of bias could not be determined. With respect to the index test, except for the two studies (15, 18), there was an ambiguous risk of bias in the remaining studies (10–14, 16, 17), and this is because there were no records of whether PET/CT was blinded to the reference criteria. With regards to reference criteria, several studies did not record whether the interpretation of reference standard results was made without understanding the results of the indicator tests (11, 14, 16), but all the studies used pathological biopsy as reference criteria and were therefore considered as low risk. Regarding the flow and timing, we believed that there is a high risk because one study did not include all enrolled patients in the analysis (12).

### Accuracy evaluation of ^68^Ga-PSMA PET/CT

**Figure 3:**
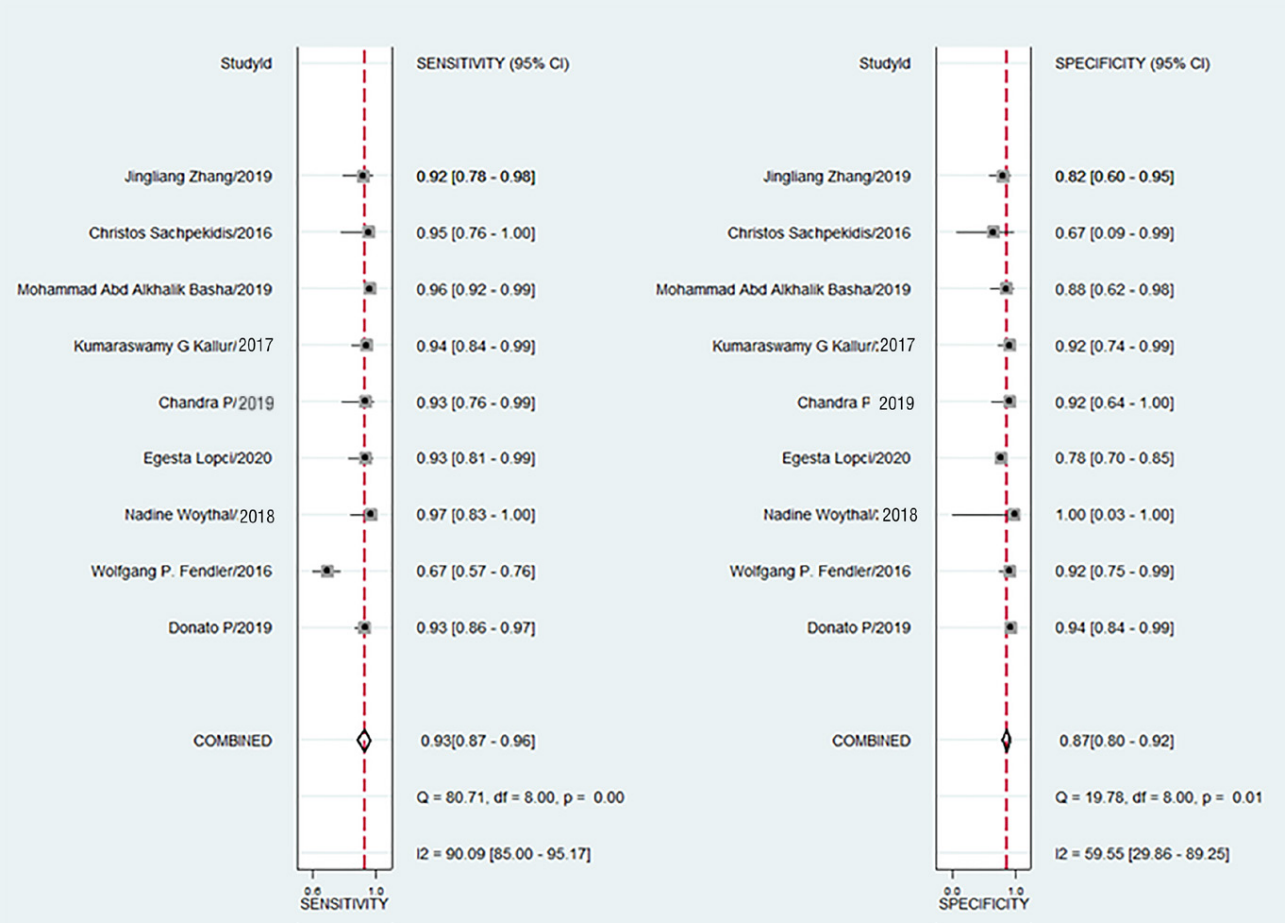
Coupled forest plots of the combination sensitivity and specificity of the inclusion study. Numbers are pooled estimates of the 95% CIs in parentheses. The lower right corner provides the corresponding statistics of heterogeneity. The horizontal lines represent the 95%CIs. CI=confidence interval. I2=heterogeneity.

**Figure 4:**
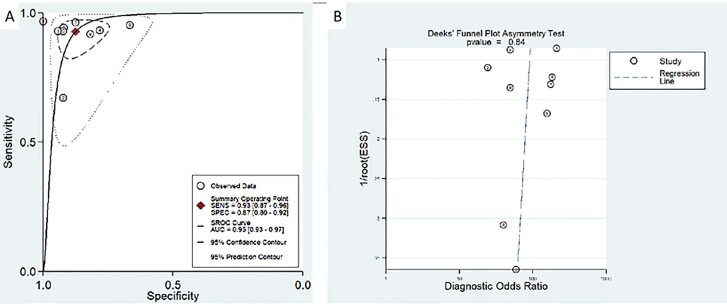
A) Hierarchical summary receiver operating characteristic curve for the diagnostic performance of 68Ga-PSMA PET/CT in patients with primary prostate cancer. B) Deeks’ funnel plot was used for each patient analysis. The p value was 0.44>0.05, indicating a low possibility of publication bias. ESS=effective sample size. AUC=area under the curve; SROC=summary receiver operating characteristic; 68Ga-PSMA=Gallium (68) labeled prostate-specific membrane antigen; PET/CT=Positron emission computed tomography/computed tomography.

Of the 9 studies, 2 studies did not record the evaluation by ^68^Ga-PSMA PET/CT (15, 16), and the other studies evaluated the positive PSMA uptake of PET/CT further by quantitative comparative analysis of SUVmax value of suspicious lesions and SUV uptake value of normal PSMA biodistribution region. The sensitivity and specificity of ^68^Ga-PSMA PET/CT in diagnosing prostate cancer in 9 studies are shown in Figure-3. The sensitivity and specificity ranges of 9 enrolled studies were 67-97% and 67%-100%, respectively. The Higgins I2 statistics showed heterogeneity in terms of sensitivity and specificity were (90.09 [95%CI, 85.00-95.17) and (59.55 [95%CI, 29.86-89.25). The pooled sensitivity and specificity in all 9 studies were 93% (95%CI=0.87-0.96) and 87% (95%CI=0.80-0.92), and the positive likelihood ratio, negative likelihood ratio and diagnostic advantage ratio were 7.4 (95%CI, 4.6-11.9), 0.08 (95%CI, 0.05-0.15) and 89 (95%CI, 42-187), respectively. The summary receiver operating characteristic (SROC) curve of the included studies is shown in Figure- 4A. A significant difference was observed between the 95% confidence interval (CI) and the prediction interval, further indicating heterogeneity among studies within this group. The area under the SROC curve was 0.95, wherein the 95%CI was 0.93-0.97. Deeks’ funnel plot is presented in Figure-4B. The P value of slope coefficient was 0.84, which was greater than 0.05, and so it is considered as low possibility of publication bias.

### Predictors of ^68^Ga-PSMA PET/CT positivity

The relationship between ^68^Ga-PSMA PET/CT positive and other factors in patients, such as PSA level, patient stage, GS, etc., was explored in 9 included studies. Some of these studies suggested that the positive diagnosis of prostate cancer by ^68^Ga-PSMA PET/CT showed association with PSA level, Gleason score and SUVmax (10, 15–18). Sachpekidis et al. (17) have found that the PSA level in prostate cancer patients showed significant correlation with the mean standard uptake value (SUVmean) and SUVmax of ^68^Ga-PSMA uptake in tumor tissues (r=0.6, r=0.57, respectively), and the Gleason score remained weak but showed marked correlation with SUVmean (r=0.33) and SUVmax (0.28). Furthermore, Lopci et al. (12) have concluded that SUVmax and SUVratio of ^68^Ga-PSMA showed obvious correlation with the accuracy of clinical diagnosis of prostate cancer in a study conducted on a total of 168 tumor tissue fragments. Moreover, Kallur et al. (14) have found that the accumulation of ^68^Ga-PSMA in tumor tissues was gradually increased with increasing prostate volume and Gleason score, but showed no distinct correlation. In another study conducted by Woythal et al. (11) on diagnostic performance of ^68^Ga-PSMA PET/CT for primary prostate cancer revealed that the SUVmax of tumor tissue was evidently higher in patients with Gleason score ≥8 than in patients with Gleason score <8, but showed no confirmed and evident correlation.

### Exploration of heterogeneity

Meta-regression analysis results show that there are significant heterogeneity in Gleason score, and in the uptake of ^68^Ga-PSMA SUVmax in the tumor tissues of prostate cancer patients (P<0.01). To be specific, the sensitivity of prostate cancer patients with Gleason score ≥8 (0.95 [95%CI 0.93-0.98]) was slightly higher than those with PSA<8 (0.93 [95%CI 0.90-0.97]), but in contrast, the specificity of the former was 0.79 ([95%CI 0.73-0.86]), which was significantly lower than that of the patients with Gleason score <8 (0.92 [95%CI 0.87 - 0.98]), and the difference was statistically significant (P <0.01). In addition, the sensitivity of mean SUVmax ≥10 (0.88 [95%CI 0.75-1.00]) was slightly lower than that of mean SUVmax <10 (0.93 [95%CI 0.86-1.00]), and the specificity of the former (0.88 [95%CI 0.73-1.00]) was greater than that of the latter (0.80 [95%CI 0.73-0.88]), but the difference between the two showed no statistical significance (P=0.26 and P=0.82, respectively).

**Figure 5:**
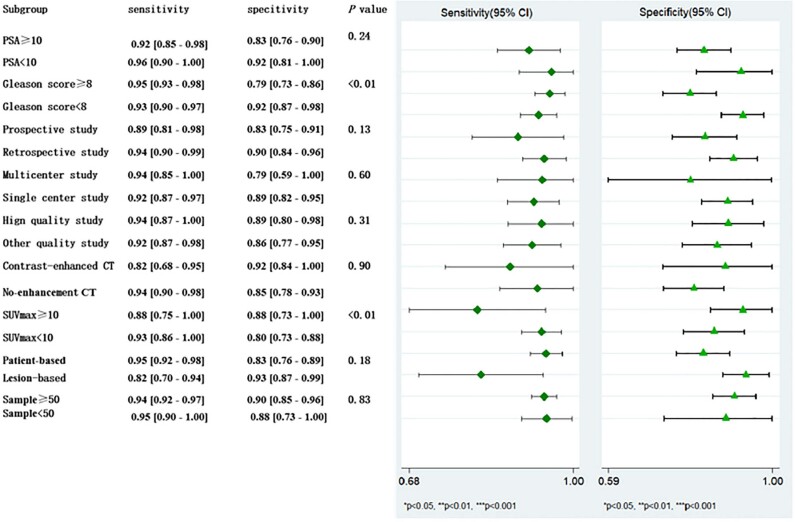
Forest plot of the sensitivity analysis, showing estimates of the pooled sensitivity and specificity of the various subgroups and the corresponding 95%CI.

The forest plot of the sensitivity analysis is presented in Figure-5. In addition to low pooled sensitivity estimates shown in both subgroups - contrast - enhanced CT techniques and based on lesion analysis - the sensitivity estimates were comparable in most of the remaining subgroups, and their pooled sensitivity ranged from 0.88 to 0.96. Nevertheless, the pooled specificity estimates were relatively low in several subgroup analyses, specifically in those subgroups that included multicenter studies (0.79 [95%CI, 0.59-1.00]), Gleason score of ≥8(0.79 [95%CI, 0.73-0.86]), and SUVmax of <10 (0.80 [95%CI, 0.73-0.88]), and this might be due to smaller sample sizes, as only two of the 9 included studies were included in the analysis of multicenter studies and Gleason score ≥8. Moreover, only 3 of the 9 studies were prospective, thus showing an estimated specificity of 0.83 with a 95%CI of 0.81-0.98.

## DISCUSSION

Prostate cancer is the most common malignancy in men after lung cancer, with nearly 430.000 new cases and more than 360.000 deaths reported each year worldwide (19). There are no specific clinical manifestations in the early stage of prostate cancer, and many patients have metastasized at the time of first diagnosis and lost the best time for treatment, leading to treatment failure. Therefore, finding effective diagnostic methods and indicators for early diagnosis of prostate cancer is the key to improve the cure rate as well as the survival rate (20, 21). Imaging examination plays an important role in the clinical diagnosis and staging of malignant tumors. However, traditional imaging examination is limited due to reduced accuracy of early diagnosis of prostate cancer (22). ^68^Ga-PSMA PET/CT, a non-invasive examination technology, has ideal application prospects, provides diagnostic performance as that of traditional imaging examination, and at the same time can detect distant metastasis, achieving “one-stop” detection efficiency and achieving early diagnosis and treatment, finally improving the prognosis of patients (23, 24). Meanwhile, ^68^Ga can be obtained by leaching with gallium germanium generator, so it is easy to produce, low cost, easy to label, and conforms to GMP (Good Manufacturing Practice) production requirements, so it is easy to clinical conversion (25). Meta-analyses have confirmed that ^68^Ga-PSMA PET/CT had dominant advantages in detecting the recurrence and staging of prostate cancer (26, 27), and the pooled sensitivity and specificity for detecting prostate cancer recurrence was 70% and 97%, as well as the pooled sensitivity and specificity of prostate cancer staging/restaging studies was 92% and 94%, respectively. However, our study is the first meta-analysis to use ^68^Ga-PSMA PET/CT for the detection of primary prostate cancer. The objective of the current meta-analysis was to explore the accuracy of ^68^Ga-PSMA PET/CT in diagnosing primary prostate cancer.

Among the enrolled studies, one study showed lower sensitivity and specificity respectively, which may be related to the small number of patients included in the analysis (21 and 24 patients included, respectively). However, the pooled sensitivity and specificity of the prostate cancer detection rates in the included 9 studies in this meta-analysis were 93% and 87%, respectively, and the results of the diagnosis of primary prostate cancer suggests that ^68^Ga-PSMA PET/CT can be used as one of the main screening methods for patients suspected with prostate cancer (with increased PSA levels, elderly patients with frequent urination, urgency, progressive urinary tract drainage and other clinical symptoms), and help determine the best treatment regimen. We did not directly compare the diagnostic performance of ^68^Ga-PSMA PET/CT and MRI in primary prostate cancer. But in a recent meta-analysis on the diagnostic performance of MRI in primary prostate cancer, Liang et al. (28) have concluded that its sensitivity and specificity was 0.77 (95%CI: 0.73-0.81) and 0.81 (95%CI: 0.76-0.85), respectively. Therefore, we believed that ^68^Ga-PSMA PET/CT had more advantages in diagnosing primary prostate cancer. As our meta-analysis results were based on a combination of patient and lesion segments, there are very few research studies on ^68^Ga-PSMA PET/CT based on the lesion fragments of prostate cancer (10, 12, 15), and we only included them in the subgroup analysis instead of studying them separately. So, it is necessary to evaluate the diagnostic effects of ^68^Ga-PSMA PET/CT in prostate cancer from patient-based and lesion-based aspects in the future work.

In terms of predicting the risk factors of patients with ^68^Ga-PSMA PET/CT positive, multiple studies have confirmed that PSA levels and Gleason scores showed close correlation. The uptake of ^68^Ga-PSMA on PET/CT in patients with prostate cancer with Gleason score of ≥8 or PSA level of ≥10ng/mL was significantly higher than those in patients with prostate cancer with Gleason score of <8 or PSA level <10 (5–18), and similar results were presented in a meta-analysis study conducted by Afshar-Oromieh et al. (29) on the prediction of ^68^Ga-PSMA PET/CT for prostate cancer recurrence. Moreover, few studies have suggested that greater the prostate volume or the higher the stage of the patients with prostate cancer, the greater is the risk of predicting ^68^Ga-PSMA PET/CT positive (11, 14).

In this meta-analysis, significant heterogeneity was observed among the 9 included studies. From meta-regression analysis, it was observed that the PSA level and average SUVmax for quantitative detection of tumor tissues by PET/CT contributed to this heterogeneity. With regard to type of study design, the quality of the included study and CT techniques used in the included study (whether contrast-enhanced scanning technology is used) showed no significant heterogeneity (P >0.05). As for the scan thickness, the uptake time and dose of the tracer, only a few articles in the 9 included studies recorded their values and were therefore not included in this meta-regression analysis. Therefore, further studies might be needed to explore additional value of slice thickness and dose and uptake time of tracers in detecting primary prostate cancer. In terms of the characteristics of the included studies, whether it is a multicenter study, prospective study, and whether the sample size is greater than 50 patients showed higher sensitivity and specificity in diagnosing prostate cancer were recorded.

One of the major limitations of our meta-analysis is that there were few studies included, and many studies in the literature regarding the diagnosis of primary prostate cancer by ^68^Ga-PSMA PET/CT were excluded due to lack of effective specific data. Secondly, most of the studies included had retrospective study designs and were non-multicenter institutional studies. Another limitation is that the sample sizes in some of the studies are smaller and the inter-study heterogeneity is large, which might in turn affect the general applicability of the results.

## CONCLUSIONS

As a new radioactive tracer of PET/CT, ^68^GA-labeled PSMA ligand had good sensitivity and specificity in detecting primary prostate cancer, and showed superior diagnostic performance in both pooled as well as subgroup analysis estimates.

## References

[1] Cornford P, Bellmunt J, Bolla M, Briers E, De Santis M, Gross T (2017). EAU-ESTRO-SIOG Guidelines on Prostate Cancer. Part II: Treatment of Relapsing, Metastatic, and Castration-Resistant Prostate Cancer. Eur Urol.

[2] Loeb S, Bjurlin MA, Nicholson J, Tammela TL, Penson DF, Carter HB (2014). Overdiagnosis and overtreatment of prostate cancer. Eur Urol.

[3] Fütterer JJ, Briganti A, De Visschere P, Emberton M, Giannarini G, Kirkham A (2015). Can Clinically Significant Prostate Cancer Be Detected with Multiparametric Magnetic Resonance Imaging? A Systematic Review of the Literature. Eur Urol.

[4] Eder M, Schäfer M, Bauder-Wüst U, Hull WE, Wängler C, Mier W (2012). ^68^Ga-complex lipophilicity and the targeting property of a urea-based PSMA inhibitor for PET imaging. Bioconjug Chem.

[5] Jadvar H (2016). Is There Use for FDG-PET in Prostate Cancer?. Semin Nucl Med.

[6] Alipour R, Gupta S, Trethewey S (2017). ^68^Ga-PSMA Uptake in Combined Hepatocellular Cholangiocarcinoma With Skeletal Metastases. Clin Nucl Med.

[7] Zhang C, Guo H (2020). Clinically diagnostic value of ^68^Ga-PSMA PET/CT imaging for clear cell renal cell carcinoma. European Urology Open Science.

[8] Malaspina S, De Giorgi U, Kemppainen J, Del Sole A, Paganelli G (2018). ^68^Ga-PSMA-PET: added value and future applications in comparison to the current use of choline-PET and mpMRI in the workup of prostate cancer. Radiol Med.

[9] Whiting PF, Rutjes AW, Westwood ME, Mallett S, Deeks JJ, Reitsma JB (2011). QUADAS-2: a revised tool for the quality assessment of diagnostic accuracy studies. Ann Intern Med.

[10] Fendler WP, Schmidt DF, Wenter V, Thierfelder KM, Zach C, Stief C (2016). ^68^Ga-PSMA PET/CT Detects the Location and Extent of Primary Prostate Cancer. J Nucl Med.

[11] Woythal N, Arsenic R, Kempkensteffen C, Miller K, Janssen JC, Huang K (2018). Immunohistochemical Validation of PSMA Expression Measured by ^68^Ga-PSMA PET/CT in Primary Prostate Cancer. J Nucl Med.

[12] Lopci E, Lughezzani G, Castello A, Saita A, Colombo P, Hurle R (2020). Prospective Evaluation of ^68^Ga-labeled Prostate-specific Membrane Antigen Ligand Positron Emission Tomography/Computed Tomography in Primary Prostate Cancer Diagnosis. Eur Urol Focus.

[13] Chandra P, Chandran G, Sangeetha J (2019). Diagnostic accuracy of pre-biopsy ^68^Ga-PSMA positron emission tomography/computed tomography for detection of prostate carcinoma. Indian Journal of Nuclear Medicine.

[14] Kumaraswamy G Kallur, Prashanth G Ramachandra, Krishnappa Rajkumar, Shivakumar S Swamy, Indiresh Desai, Raghavendra M Rao (2017). Clinical utility of gallium-68 PSMA PET/CT scan for prostate cancer. Indian Journal of Nuclear Medicine.

[15] Donato P, Morton A, Yaxley J, Ranasinghe S, Teloken PE, Kyle S (2020). ^68^Ga-PSMA PET/CT better characterises localised prostate cancer after MRI and transperineal prostate biopsy: Is ^68^Ga-PSMA PET/CT guided biopsy the future?. Eur J Nucl Med Mol Imaging.

[16] Basha MAA, Hamed MAG, Hussein O, El-Diasty T, Abdelkhalek YI, Hussein YO (2019). ^68^Ga-PSMA-11 PET/CT in newly diagnosed prostate cancer: diagnostic sensitivity and interobserver agreement. Abdom Radiol (NY).

[17] Sachpekidis C, Kopka K, Eder M, Hadaschik BA, Freitag MT, Pan L (2016). ^68^Ga-PSMA-11 Dynamic PET/CT Imaging in Primary Prostate Cancer. Clin Nucl Med.

[18] Zhang J, Shao S, Wu P, Liu D, Yang B, Han D (2019). Diagnostic performance of ^68^Ga-PSMA PET/CT in the detection of prostate cancer prior to initial biopsy: comparison with cancer-predicting nomograms. Eur J Nucl Med Mol Imaging.

[19] Bray F, Ferlay J, Soerjomataram I, Siegel RL, Torre LA, Jemal A (2018). Global cancer statistics 2018: GLOBOCAN estimates of incidence and mortality worldwide for 36 cancers in 185 countries. CA Cancer J Clin.

[20] Johnston AW, Longo TA, Davis LG, Zapata D, Freedland SJ, Routh JC (2020). Bone scan positivity in non-metastatic, castrate-resistant prostate cancer: external validation study. Int Braz J Urol.

[21] Vidal M, Delgado A, Martinez C, Correa JJ, Durango IC (2020). Overall survival prediction in metastatic castration-resistant prostate cancer treated with radium-223. Int Braz J Urol.

[22] Maresca KP, Hillier SM, Femia FJ, Keith D, Barone C, Joyal JL (2009). A series of halogenated heterodimeric inhibitors of prostate specific membrane antigen (PSMA) as radiolabeled probes for targeting prostate cancer. J Med Chem.

[23] Sandler KA, McClelland S, Degnin C, Chen Y, Mitin T (2019). Dramatic polarization in genitourinary expert opinions regarding the clinical utility of positron emission tomography (PET) imaging in prostate cancer. Int Braz J Urol.

[24] Leitsmann C, Thelen P, Schmid M, Meller J, Sahlmann CO, Meller B (2019). Enhancing PSMA-uptake with androgen deprivation therapy - a new way to detect prostate cancer metastases?. Int Braz J Urol.

[25] Cuccurullo V, di Stasio GD, Evangelista L, Ciarmiello A, Mansi L (2018). Will ^68^Ga PSMA-radioligands be the only choice for nuclear medicine in prostate cancer in the near future? A clinical update. Rev Esp Med Nucl Imagen Mol.

[26] Lin CY, Lee MT, Lin CL, Kao CH (2019). Comparing the Staging/Restaging Performance of ^68^Ga-Labeled Prostate-Specific Membrane Antigen and 18F-Choline PET/CT in Prostate Cancer: A Systematic Review and Meta-analysis. Clin Nucl Med.

[27] Corfield J, Perera M, Bolton D, Lawrentschuk N (2018). ^68^Ga-prostate specific membrane antigen (PSMA) positron emission tomography (PET) for primary staging of high-risk prostate cancer: a systematic review. World J Urol.

[28] Liang Z, Hu R, Yang Y, An N, Duo X, Liu Z (2020). Is dynamic contrast enhancement still necessary in multiparametric magnetic resonance for diagnosis of prostate cancer: a systematic review and meta-analysis. Transl Androl Urol.

[29] Afshar-Oromieh A, Avtzi E, Giesel FL, Holland-Letz T, Linhart HG, Eder M (2015). The diagnostic value of PET/CT imaging with the (68)Ga-labelled PSMA ligand HBED-CC in the diagnosis of recurrent prostate cancer. Eur J Nucl Med Mol Imaging.

